# Native Agarose Gels and Contact Blotting as Means to Optimize the Protocols for the Formation of Antigen–Ligand Complexes

**DOI:** 10.3390/bioengineering10101111

**Published:** 2023-09-22

**Authors:** Claudia D’Ercole, Ario de Marco

**Affiliations:** Laboratory of Environmental and Life Sciences, University of Nova Gorica, Vipavska Cesta 13, P.O. Box 301, SI-5000 Nova Gorica, Slovenia; claudia.dercole@ung.si

**Keywords:** protein complexes, adhirons, agarose native gels, fluorescent immunoreagents

## Abstract

Background: Protein complexes provide valuable biological information, but can be difficult to handle. Therefore, technical advancements designed to improve their manipulation are always useful. Methods: We investigated the opportunity to exploit native agarose gels and the contact blot method for the transfer of native proteins to membranes as means for optimizing the conditions for obtaining stable complexes. As a simple model of protein–protein interactions, an antigen–ligand complex was used in which both proteins were fused to reporters. Results: At each step, it was possible to visualize both the antigen, fused to a fluorescent protein, and the ligand, fused to a monomeric ascorbate peroxidase (APEX) and, as such, a way to tune the protocol. The conditions for the complex formation were adapted by modifying the buffer conditions, the concentration of the proteins and of the cross-linkers. Conclusions: The procedure is rapid, inexpensive, and the several detection opportunities allow for both the monitoring of complex stability and the preservation of the functionality of its components, which is critical for understanding their biomedical implications and supporting drug discovery. The overall protocol represents a handy alternative to gel filtration, uses very standard and ubiquitous equipment, and can be implemented rapidly and without specific training.

## 1. Introduction

Protein interactions and their assembling into complexes play a key role in the regulation of biological activities, and consequently, the assessment of the qualitative and quantitative parameters regulating the formation of such complexes has been addressed by adopting several methodologies [1,2,3]. Such protocols can be extremely reliable in recovering even variants of protein–protein complexes [4] and, in principle, could also be applicable to characterize ligands of different natures used to bind to protein targets and/or affect protein–protein interactions. The drawback of these methods is that they often require cumbersome optimization steps, are technically demanding, need high quality reagents and sophisticated equipment and the operators must undergo specific training. All this makes them not particularly suitable for preliminary screening aimed at optimizing the protocol conditions, such as the buffers or the cross-linkers used in different applications, or the tuning of other factors that can contribute to improving the experimental output. In this context, it would be particularly beneficial to have a handy solution for preliminary protocol evaluation.

We were interested in a peculiar kind of complex, namely those formed by the interaction between (unknown) antigens and their specific ligands, with the perspective of isolating the complex for downstream analysis by mass spectrometry and final antigen identification. This de-orphaning process is meaningful any time that a bite is used to capture unknown protein cognates, and several alternative strategies that share the aim of identifying the molecular targets by means of specific proximity labeling have been proposed [5,6,7,8]. No matter what protocol will be used, the researcher will need to optimize the experimental conditions to adapt them to the specific context. Therefore, we looked for a rapid and inexpensive method with a simple read-out to assess the experimental factors affecting the quality of the ligand–antigen complex formation process that is instrumental in obtaining effective immunoprecipitation and successive target protein processing. We oriented our attention towards methodologies that preserve the protein functionality because this condition allows either direct visualization (as in the case of fluorescent proteins) or indirect detection by means of enzymatic activities.

Recent advances in the field of native agarose electrophoresis and blotting [9,10,11,12] demonstrate that this technique can be more convenient than alternative electrophoretic techniques to separate and evaluate antibodies as well as macromolecular complexes in their native state [13]. We successfully exploited it to characterize enzyme isoforms by in-gel activity staining [14]. Starting from this background, we investigated the possibility of combining native agarose electrophoresis and contact blotting for the evaluation of ligand–protein complexes and for identifying conditions that improve the stabilization and preservation of such structures. This achievement is instrumental for de-orphaning scopes as well as for preparing material that will undergo downstream structural and characterization studies, such as NMR, cryo-electron microscopy, or deuterium exchange mass spectrometry analyses [15]. In particular, to demonstrate the versatility of this approach, we used the green fluorescent protein mClover3 fused to one of the complex components, a condition that allowed for their direct detection without the requirement of staining procedures. In addition, the fusion of the cognate with the modified ascorbate peroxidase APEX [16] allowed for the construct localization after the enzyme-dependent development of colored substrate.

## 2. Materials and Methods

### 2.1. Protein Production

The constructs corresponding to the fusion proteins SpyCatcher-mClover3 (SC), adhiron AntiSpyCatcher-APEX (ACA), and anti-CD63 nanobody-SpyTag (ST) were cloned into the pET-14b vector to express constructs with a C-terminal 6xHis tag. SC was transformed in *E. coli* BL21(D3) cells that were grown at 37 °C and 210 rpm until the OD_600_ reached 0.8; then, the temperature was decreased to 20 °C, the recombinant protein expression was induced with 0.1 mM IPTG, and the cells were harvested after overnight culture by centrifugation (15 min at 6500× *g*). ACA and ST were transformed in *E. coli* BL21(D3) strain co-expressing a sulfhydryl oxidase (SOX, [17]), which allowed for the formation of disulfide bonds. Bacteria were initially grown at 37 °C and 210 rpm, then 0.2% (*w*/*v*) of arabinose was added at the OD_600_ of 0.4 to induce SOX expression. The temperature was decreased to 20 °C, and the recombinant protein expression was induced after further 30 min (OD_600_ around 0.8) by adding 0.1 mM IPTG. The bacterial pellet was recovered after overnight culture as described above. All pelleted cells were lysed in 4 volumes of 50 mM Tris-HCl pH 8.0, 500 mM NaCl, 5 mM MgCl_2_ by alternating three cycles of freezing/thawing. Lysates were sonicated on ice (6×, amplitude 80%, 1 min pulse, 1 min off), incubated for 30 min at room temperature in the presence of DNAse (33 U/mL) and lysozyme (100 μg/mL), and finally centrifuged at 4 °C (30 min at 13,000× *g*). Proteins were purified by fast liquid chromatography (FPLC) using an ÄKTA pure™ system. The supernatants were injected at a flow rate of 1 mL/min into a Talon Hi-Trap column (Cytiva—Marlborough, MA, USA), previously equilibrated in 50 mM Tris-HCl pH 8.0, 500 mM NaCl, 15 mM imidazole. After column washing in the same buffer, the bound proteins were eluted in 50 mM Tris-HCl pH 8.0, 500 mM NaCl, 500 mM imidazole. The samples were desalted in PBS/glycerol 10%, pH 7.4, by means of a Hi-Trap Desalting column (Cytiva) and evaluated by gel filtration (Superdex 200 10/300 GL, Cytiva) and SDS-PAGE. Proteins were quantified by recording the values of UV absorption at 280 nm and calculating the concentrations by using their specific molar extinction coefficient. A colorimetric assay was performed to assess the enzymatic activity of APEX fused to the adhiron (ACA construct). ACA was incubated with 50 μM of Amplex™ UltraRed (Thermo Fisher, Waltham, MA, USA) according to the producer’s specifications, and the reaction efficiency was quantified recording the emitted fluorescence at 595 nm. The complex formation was induced by incubating equimolar amounts of the protein partners in HEPES or PBS buffers.

### 2.2. Cross-Linking Strategies

Equimolar amounts of SC and ACA were covalently linked using either glutaraldehyde or disuccinimidyl suberate (DSS). Since they have almost identical molecular weight, in the case of glutaraldehyde, 10 μg of both antigen and ligand were resuspended in 20 mM HEPES, pH 7.5, to a final volume of 100 μL to which 5 μL of an instantly prepared 2.3% glutaraldehyde solution were added. In the case of DSS treatment, different amounts of both proteins (5, 10, 20 μg) were diluted in 100 μL of either 20 mM HEPES, pH 7.5, or PBS, pH 7.4, containing either 1 or 2 mM of DSS. In all cases, the protein components were first incubated at 4 °C for 120 min under constant rotation to induce the complex formation. Next, the cross-linkers were added and samples were incubated at room temperature under constant agitation for different times to identify the optimal conditions. The reactions were quenched by adding 50 mM Tris-HCl, pH 8.0.

### 2.3. Native Agarose Gel

Agarose (1%) was dissolved in Tris-glycine buffer pH 7.5, heated, and cast onto a flat bed with a comb set in the middle. Single fusion proteins and their relative complexes were mixed (4:1 *v*/*v*) with native loading buffer (200 mM Tris HCl pH 6.8, 20% glycerol, 50 mM EDTA, 0.8 mg/mL Bromophenol Blue), and horizontal agarose native gel electrophoresis was run on ice for 40–60 min under a constant voltage of 100 V, as described before [14].

### 2.4. Contact Blotting

After native agarose electrophoresis, proteins were transferred to a nitrocellulose membrane by contact blotting, a methodology that preserves native conditions, as recently described [11]. The transfer system was composed of two thin standard filter papers, one thick Whatman paper (Cytiva GB005, Cat. No. 10426981), a nitrocellulose membrane (VWR, Cat. No. 10600001), and an agarose gel isolated through a plastic wrap from a 3 kg metal block. The filter and Whatman paper sheets as well as the nitrocellulose membrane were preliminary wet in transfer buffer (25 mM Tris, 192 mM glycine, 1% SDS, 20% methanol). The transfer sandwich was pressed by means of the metal block for 10–15 min to obtain effective protein blotting on the membrane. Blotted membranes were kept wet for downstream detection assays.

### 2.5. Protein Detection and Functionality Assays

Fluorescent proteins were visualized directly in the agarose gels using a UV transilluminator (Cleaver Scientific, Rugby, UK). Blotted membranes were stained with Ponceau S solution, and proteins were also detected by means of anti-HisTag-HRP (horseradish peroxidase) antibodies (Thermo Fisher, Cat No. MA1-80218, Waltham, MA, USA) in combination with Novex™ ECL (Invitrogen Chemiluminescent Substrate Reagent Kit, Cat No. WP20005, Waltham, MA, USA) as a reaction substrate. Membranes were imaged with a UVITEC chemiluminescence imaging system (Cambridge, UK). In situ APEX activity in agarose gels was detected in the presence of TMB (3,3′,5,5′-Tetramethylbenzidine) (VWR—Cat. No. J644-100 ML) until colored bands appeared. APEX was used to identify blotted antigen (SC) by adding ACA diluted 1:200 in PBS/5% milk in combination with a Chemiluminescent Substrate Reagent Kit (Invitrogen, Cat No. WP20005). Membranes were imaged as above. APEX activity was also measured on membranes blotted with ACA. Membrane lanes were cut and incubated with either TMB or Amplex^®^UltraRed, and APEX activity was measured by using the spectrophotometer to read the absorbance at 450 nm and the fluorescence at 595 nm for TMB and Amplex^®^UltraRed, respectively.

## 3. Results

Adhirons are synthetic ligands that can be used as substitutes for immunoglobulins to bind to and detect antigens [18]. Recently, adhirons specific for the SpyCatcher domain [19] were isolated from a synthetic phage display library, and the assessment of their biophysical characteristics confirmed the formation of a complex between the adhiron G5 and its target SpyCatcher [20]. Since only few adhiron structures are available and even fewer reports describe the interactions of complexes formed by adhirons and their targets at the molecular level, we planned to use this complex as a simple but meaningful model for assessing our optimization strategy and obtaining a final reagent suitable for NMR analysis. As a preliminary step, we wished to optimize the conditions for recovering a stable complex and evaluated the possibility of using native agarose gels and contact blotting for protocol optimization, extending the applications of the original work performed by Arakawa and co-workers [12].

**Figure 1:**
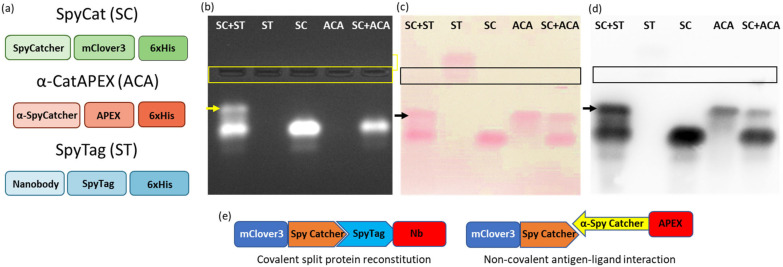
Effective protein transfer on nitrocellulose membrane by contact blotting. (**a**) Schematic representation of the constructs used in the experiment. (**b**) SpyTag (ST), SpyCatcher-mClover3 (SC), the fusion between an adhiron AntiCatcher and APEX (ACA) and their relative complexes underwent electrophoresis in agarose native gel, and the fluorescent SC construct was visualized using a transilluminator. The complex formed by SC + ST is indicated by an arrow. (**c**) After blotting on nitrocellulose membrane, proteins were stained with Ponceau S solution. (**d**) The same blotted proteins were visualized by means of anti-HisTag-HRP antibodies. (**e**) Schematic representation of the covalent complex between SC and ST (**left**) and the non-covalent interaction between SC and ACA (**right**).

**Figure 2:**
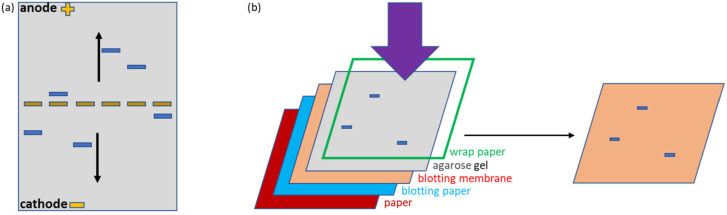
Characteristics of native agarose gel and native contact blotting. (**a**) Samples loaded in the middle of a flat native agarose gel can freely migrate, independently of their charge. (**b**) Contact blotting does not employ denaturing conditions and allows the transfer of native proteins on nitrocellulose. The membrane is placed face-to-face with the gel, and the weight on the top of the sandwich (**left**) promotes the migration of the proteins that are trapped on the membrane (**right**).

The initial experiment was designed to visualize the stability of protein complexes during migration in native agarose gel. Two systems were compared, the first corresponding to the covalent interaction between a construct formed by an irrelevant nanobody and the SpyTag (ST) and the fusion construct SpyCatcher-mClover3 (SC), the second corresponding to the non-covalent complex formed by an anti-SpyCatcher adhiron fused to APEX (ACA) and SC (Figure 1a,e). The fluorescent (mClover3) and enzymatic partners (APEX) were chosen to simplify the protein visualization. Furthermore, all constructs possessed a HisTag suitable for affinity purification and detection. The samples were deposited in the middle of the native agarose gel. This arrangement is particularly useful because allows the migration of any protein, independently of its net charge, towards either the anode or cathode, in contrast to what happens in a conventional electrophoretic setting in which the samples are loaded on one edge and can migrate only if they have a compatible charge (Figure 2a). This complete protein distribution overview is instrumental for the recognition of variations inside the migration pattern due to interactions between single proteins. The native conditions used for electrophoresis were chosen to preserve protein functionality and, indeed, the fluorescent signal provided by the mClover3 moiety of SC samples was preserved and clearly detected in the gel using a transilluminator (Figure 1b). ST and ACA were localized after native transfer to a blot membrane (Figure 2b) by Ponceau red staining (Figure 1c) and by Western blot (WB), exploiting an anti-His-tag antibody fused to HRP (Figure 1d). ST migrated towards the anode, SC and ACA towards the cathode (Figure 1c). The His-Tag of ST was apparently not accessible to the corresponding antibodies and remained undetected in WB (Figure 1d). SC and ACA apparently dissociated during migration, since no new complex-specific band appeared in Figure 1c,d. In contrast, SC and ST were able to reconstitute the covalent bond, and a new band, corresponding to the complex, became visible in the gel (indicated by an arrow in Figure 1b–d). Next, we investigated the possibility of stabilizing the SC-ACA complex by inducing intermolecular linkages between the two components (Figure S1). Since they have comparable mass, the same amount of protein was used for both samples. It is important to assess the effect of such a step because of its potential applications. For instance, creating a covalent linkage can be very useful to conserve complexes between a known ligand and an unknown antigen and purify them by means of tags fused to the recombinant ligand. We initially assessed glutaraldehyde as a cross-linker because it is inexpensive and highly reactive. The drawback of this reagent is its low substrate specificity (it can react with as many as six different amino acids) and its propensity to polymerize in aqueous solutions [21]. In the case of our protein system, the use of glutaraldehyde resulted in the formation of apparently heterogeneous products (large smears visible in the gel) with characteristics that changed progressively, according to the length of the incubation time used to promote the component cross-reaction (Figure S1b). Since the mClover3 fluorescence was not significantly reduced by the treatment, the glutaraldehyde-dependent polymerization effects were visible directly in the native gel, without the necessity to blot and visualize the proteins on the membrane (Figure S1), as we did to show a control of the protein signal detected in the gel.

**Figure 3:**
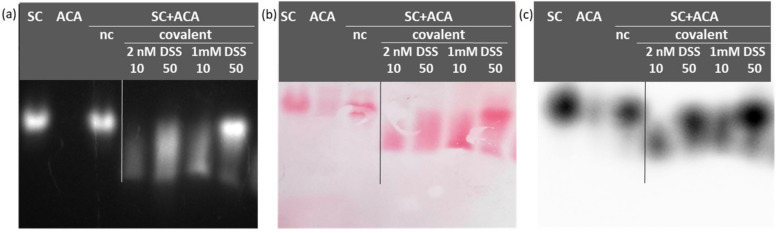
Detection of covalent complex obtained after DSS treatment. The DSS-dependent formation of covalent interactions between SC and ACA was evaluated at different conditions and compared with the results obtained in the absence of cross-linker (nc—not covalent). Either 10 or 50 µg of the reagents (1:1 in terms of molarity) was allowed reacting with 1 or 2 mM DSS before electrophoresis and visualized by fluorescence using a transilluminator (**a**), by Ponceau S staining after blotting on nitrocellulose (**b**), and by chemiluminescence signal using anti-HisTag-HRP antibodies (**c**).

These preliminary results prompted us to test the non-cleavable cross-linker DSS. This alternative reagent has the advantage of being reactive only with amine groups and compatible with downstream mass spectrometry analyses. In this case, as well, the treatment preserved the fluorescence of mClover3 that was instrumental in tracing the peculiar migration of the complex with respect to the mobility of the original reagents (Figure 3a). In comparison to glutaraldehyde, the complex between SC and ACA formed in the presence of DSS was less diffuse, at least at optimized experimental conditions (Figure 3). Once the preferential buffer between PBS and HEPES (Figure S2) was selected, the most homogeneous complex sample was obtained using equimolar amounts of antigen and ligand (SC + ACA, 50 µg for each reagent) in the presence of 1 mM DSS.

**Figure 4:**
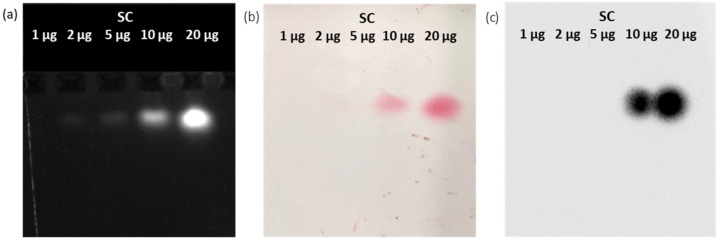
Adhiron-APEX as an effective reagent for identification of blotted antigens. Different concentrations of SC underwent electrophoresis (**a**), were transferred by contact blotting and stained with Ponceau red (**b**), and were finally stained by exploiting the APEX activity of ACA in the presence of chemiluminescent substrate (**c**).

Next, we evaluated another detection opportunity that is offered by the APEX activity of ACA. We first quantified in 10 µg the minimal amount of SC necessary for its detection in the gel (by transilluminator-detected fluorescence, Figure 4a) and after its transfer to the membrane (by Ponceau red staining, Figure 4b). Then, the SC bands on the blotted membrane were identified by ACA addition (Figure 4c), since this reagent possesses a ligand moiety (the anti-SC adhiron) that can specifically bind to its cognate SC, whereas the APEX moiety can catalyze a chemiluminescent reaction in the presence of TMB. The very intense signals demonstrated that adhiron-APEX fusion molecules such as ACA can be used as an effective alternative to antibody-HRP reagents.

**Figure 5:**
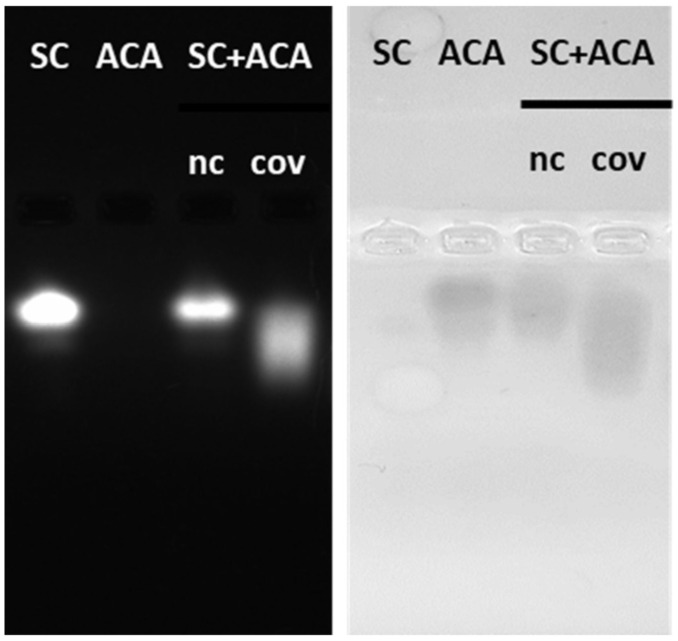
Detection of APEX peroxidase activity in native agarose gel. SC, ACA and their complex underwent electrophoresis in the absence (nc) or presence (cov—covalent) of 1 mM DSS used as a cross-linker. UV transilluminator was used to detect the fluorescent mClover3 moiety of SC (**left**), then the native agarose gel was incubated in TMB (peroxidase substrate) and the APEX activity allowed the identification of ACA and its covalent complex (**right**).

Altogether, these results show the usefulness of the approach to set the experimental parameters in the case of known samples. Nevertheless, we wished to prove the possibility of using native conditions for both the electrophoresis and the transfer steps to direct in situ (on blotted membranes) detection of bands corresponding to any complex formed by a recombinant ligand and an unknown antigen. Namely, the known biochemical features of the recombinant ligand (the bite) will be used to trace the unknown captured antigen. In our simplified model composed of SC and ACA, ACA activity could be detected directly in native gels using TMB as the substrate since APEX successfully oxidized it into its bluish product (Figure S3). The analysis of APEX activity also allowed confirming that its enzymatic activity was lost after glutaraldehyde-mediated cross-linking of SC with ACA (Figure S3b). In contrast, APEX activity was preserved when the complex was cross-linked using DSS (Figure 5). We could initially also determine that APEX activity remained detectable after its transfer to a nitrocellulose membrane. However, differently from the experiment described above in which ACA was used as a soluble WB reagent for antigen visualization, the peroxidase activity of blotted APEX did not result in an in situ protein visualization (Figure S4), but rather in the staining of the buffer used for the reaction. Therefore, another protocol was envisaged. First, the protein bands stained by Ponceau red were cut and then incubated singularly in the presence of the peroxidase substrates TMB and AmplexUltraRed. All the samples containing ACA, alone or in complex with SC, showed APEX activity and successfully oxidized the substrates, confirming that the enzyme functionality was preserved after blotting and after complexation of the ligand with its antigen (Figure S4d). Blotted SC can be considered as a negative control and indeed had no peroxidase activity.

## 4. Discussion

Native electrophoresis performed using agarose gels [9,11] has been successfully exploited to study protein conformational modifications and aggregation [22,23], but the method appeared to us suitable for the characterization of protein complexes as well. The study of protein complexes allows the understanding of the molecular regulation of biological processes [1], and therefore, it is particularly meaningful to develop protocols that can preserve the complex assembly during the purification steps. Furthermore, it is necessary that the complex elements maintain their native conformation to obtain reliable biological information and avoid misleading conclusions due to the presence of artifacts. In this perspective, the possibility of following the quality of protein complexes along the purification process in a simple and fast way is critical to speed up its optimization. We used here a simple model of a protein complex, the one constituted by an antigen–antibody pair, to evaluate an approach based on the use of native migration and transfer steps that enables the selection of the experimental conditions necessary for preserving the integrity of the complex and the functionality of the single components. The model is relevant for itself when applied to de-orphaning processes, namely the identification of unknown antigens bound by ligands isolated by means of blind panning of large libraries or by co-precipitation experiments aimed at isolating components of interactomes [24,25,26]. We plan to improve the transfer efficiency and consequently the limit of detection to apply this procedure for the identification of receptors isolated directly from cell membranes.

We designed specific reagents to evaluate all the opportunities offered by the approach, although some of these conditions will be not always suitable in biological applications. Nevertheless, fluorescent proteins are often fused to complex sub-units to allow imaging or functional knock-down [27,28], and small recombinant ligands such as nanobodies or adhirons can be easily produced as fusions with different tags with enzymatic activities [29]. The first advantage of using native agarose gels as the electrophoretic step is that this system enables the loading of the samples in the middle of a flat slab, and this solution is compatible with the migration of any protein construct, independently of its isoelectric point, towards one of the two electric poles. This means that no information is lost, as happens in conventional electrophoretic settings in which only proteins with a defined charge will move through the gel. As a consequence of protein–protein interactions, the migration pattern will change and will be easily detectable. When any fluorescent protein is available, a simple observation of the gel with a transilluminator will be sufficient. Otherwise, we showed that the enzymatic activity of a fusion partner such as APEX will effectively catalyze a color reaction suitable to evidence the corresponding protein bands directly in the gel (Figure 5). The native transfer blotting obtained by simple contact is not as efficient as the conventional WB, but preserving the protein functionality can, again, provide a further opportunity to identify the proteins of interest. In our experiments, we successfully exploited Ponceau red staining or the peroxidase activity of APEX to spot the different proteins, but failed to detect ST by conventional WB using a commercial anti-HisTag antibody (Figure 1). There are several reasons for which antibodies do not work in a specific technique [30], from the poor accessibility of the epitope to its loss under specific experimental conditions [11]. In consideration of these shortcomings, the example shows how meaningful is to have alternative imaging options to visualize the target proteins, last but not least, the availability of an adhiron-APEX construct (ACA) that can be used as an effective substitute for conventional WB reagents made of a fusion between antibodies and HRP (Figure 4). It should be underlined that ACA can be produced very cost-effectively in bacteria, whereas commercial antibody-HRP products are notoriously expensive.

Apart from the issues discussed above, which are suitable for any protein, there are specific issues to consider when we deal with protein complexes. These can have very variable strength and can be easily dissolved in the absence of stabilizing conditions that must be identified. The screening of different buffers is the first logical step, and our approach is particularly indicated for this test since several samples can be assessed in parallel and both gel migration and contact blotting are extremely rapid. In our example, we compared 6 samples in 90 min, but more samples (at least 12) can be analyzed in the same time using a larger slab. This speed outperforms complex-stability evaluation protocols based on gel filtration. This is true also for the next parameter we optimized, namely the conditions to use for cross-linking the complex components. In this case, it was important to evaluate three parameters: (i) homogeneity of the resulting complex; (ii) conservation of the native structure; (iii) effect of experimental factors (sample and cross-linker concentrations and their combination with buffers). All these parameters were easily assessable using our setting by which we could directly visualize and compare the effect of the different treatments on the same slab (Figure 3), avoiding potential misleading indications due to suboptimal reproducibility of any experimental step. The process is clearly simpler than methods requiring primary and secondary antibodies with several steps of incubation and washing. Furthermore, native gel electrophoresis seems more suitable than analytical gel filtration to appreciate the complexity of aggregation/large polymerization since there is not an exclusion limit that compresses all the molecular species larger than that dimension in a unique peak.

## 5. Conclusions

The experimental results reported in this work demonstrate that native agarose gel and (native) contact blotting are both meaningful options to quickly evaluate and optimize the parameters for preserving functional protein complexes in a format suitable for downstream characterization. The process is critically simplified by fusing reporter proteins to the reagents used as bites. Such reporters must be either directly detectable, such as fluorescent proteins, or should catalyze enzymatic reactions. APEX seems particularly suitable since there are several commercial substrates available for peroxidases, and in contrast to most of the other enzymes belonging to this family, it is simple to produce in a functional form in bacteria. The setting is inexpensive, and probably any lab has already all the necessary components and the expertise to organize the experiments.

## Data Availability

Data will be available on request.

## Supplementary Materials

The following supporting information can be downloaded at: https://www.mdpi.com/article/10.3390/bioengineering10101111/s1, Figure S1: Detection of ACA-SC covalent complex obtained after glutaraldehyde treatment; Figure S2: Buffer optimization for DSS-dependent protein complex cross-linking; Figure S3: Detection of APEX peroxidase in native agarose gel; Figure S4: Quantitative evaluation of APEX activity after its blotting on nitrocellulose membrane.

## References

[1] Puig O., Caspary F., Rigaut G., Rutz B., Bouveret E., Bragado-Nilsson E., Wilm M., Séraphin B. (2001). The tandem affinity purification (TAP) method: A general procedure of protein complex purification. Methods.

[2] Van Nostrand E.L., Pratt G.A., Shishkin A.A., Gelboin-Burkhart C., Fang M.Y., Sundararaman B., Blue S.M., Nguyen T.B., Surka C., Elkins K. (2016). Robust transcriptome-wide discovery of RNA-binding protein binding sites with enhanced CLIP (eCLIP). Nat. Methods.

[3] Weaver S.D., Schuster-Little N., Whelan R.J. (2022). Preparative capillary electrophoresis (CE) fractionation of protein digests improves protein and peptide identification in bottom-up proteomics. Anal. Methods.

[4] Cappelletti V., Hauser T., Piazza I., Pepelnjak M., Malinovska L., Fuhrer T., Li Y., Dörig C., Boersema P., Gillet L. (2021). Dynamic 3D proteomes reveal protein functional alterations at high resolution in situ. Cell.

[5] Subbotin R.I., Chait B.T. (2014). A pipeline for determining protein-protein interactions and proximities in the cellular milieu. Mol. Cell. Proteom..

[6] Paek J., Kalocsay M., Staus D.P., Wingler L., Pascolutti R., Paulo J.A., Gygi S.P., Kruse A.C. (2017). Multidimensional Tracking of GPCR Signaling via Peroxidase-Catalyzed Proximity Labeling. Cell.

[7] Ariotti N., Rae J., Giles N., Martel N., Siereck I.E., Gambin Y., Hall T.E., Parton R.G. (2018). Ultrastructural localisation of protein interactions using conditionally stable nanobodies. PLoS Biol..

[8] Hedl T.J., San Gil R., Cheng F., Rayner S.L., Davidson J.M., De Luca A., Villalva M.D., Ecroyd H., Walker A.K., Lee A. (2019). Proteomics Approaches for Biomarker and Drug Target Discovery in ALS and FTD. Front. Neurosci..

[9] Li C., Arakawa T. (2019). Agarose native gel electrophoresis of proteins. Int. J. Biol. Macromol..

[10] Li C., Akuta T., Nakagawa M., Sato T., Shibata T., Maruyama T., Okumura C.J., Kurosawa Y., Arakawa T. (2020). Agarose native gel electrophoresis for characterization of antibodies. Int. J. Biol. Macromol..

[11] Akuta T., Maruyama T., Sakuma C., Nakagawa M., Tomioka Y., Entzminger K., Fleming J.K., Sato R., Shibata T., Kurosawa Y. (2022). A New Method to Characterize Conformation-Specific Antibody by a Combination of Agarose Native Gel Electrophoresis and Contact Blotting. Antibodies.

[12] Arakawa T., Nakagawa M., Tomioka Y., Sakuma C., Li C., Sato T., Sato R., Shibata T., Kurosawa Y., Akuta T. (2022). Gel-electrophoresis based method for biomolecular interaction. Methods Cell Biol..

[13] Wittig I., Schägger H. (2009). Native electrophoretic techniques to identify protein-protein interactions. Proteomics.

[14] Oloketuyi S., Annovi G., de Marco A. (2020). Peroxidase zymograms obtained by agarose native gel electrophoresis have unmet resolution and completeness. Int. J. Biol. Macromol..

[15] James E.I., Murphree T.A., Vorauer C., Engen J.R., Guttman M. (2022). Advances in Hydrogen/Deuterium Exchange Mass Spectrometry and the Pursuit of Challenging Biological Systems. Chem. Rev..

[16] Sherwood L.J., Hayhurst A. (2022). Visualizing Filoviral Nucleoproteins Using Nanobodies Fused to the Ascorbate Peroxidase Derivatives APEX2 and dEAPX. Methods Mol. Biol..

[17] Veggiani G., de Marco A. (2011). Improved quantitative and qualitative production of single-domain intrabodies mediated by the co-expression of Erv1p sulfhydryl oxidase. Protein Expr. Purif..

[18] Tiede C., Tang A.A., Deacon S.E., Mandal U., Nettleship J.E., Owen R.L., George S.E., Harrison D.J., Owens R.J., Tomlinson D.C. (2014). Adhiron: A stable and versatile peptide display scaffold for molecular recognition applications. Protein Eng. Des. Sel..

[19] Reddington S.C., Howarth M. (2015). Secrets of a covalent interaction for biomaterials and biotechnology: SpyTag and SpyCatcher. Curr. Opin. Chem. Biol..

[20] D’Ercole C., De March M., Veggiani G., Oloketuyi S., Svigelj R., de Marco A. A new Adhiron library allows expanding the biological applications of small synthetic binders. Biomolecules.

[21] Köhler M., Neff C., Perez C., Brunner C., Pardon E., Steyaert J., Schneider G., Locher K.P., Zenobi R. (2018). Binding Specificities of Nanobody Membrane Protein Complexes Obtained from Chemical Cross-Linking and High-Mass MALDI Mass Spectrometry. Anal. Chem..

[22] Sato R., Tomioka Y., Sakuma C., Nakagawa M., Kurosawa Y., Shiba K., Arakawa T., Akuta T. (2023). Detection of concentration-dependent conformational changes in SARS-CoV-2 nucleoprotein by agarose native gel electrophoresis. Anal. Biochem..

[23] Tomioka Y., Sato R., Takahashi R., Nagatoishi S., Shiba K., Tsumoto K., Arakawa T., Akuta T. (2023). Agarose native gel electrophoresis analysis of thermal aggregation controlled by Hofmeister series. Biophys. Chem..

[24] Even-Desrumeaux K., Chames P. (2012). Phage display and selections on cells. Methods Mol. Biol..

[25] Crépin R., Gentien D., Duché A., Rapinat A., Reyes C., Némati F., Massonnet G., Decaudin D., Djender S., Moutel S. (2017). Nanobodies against surface biomarkers enable the analysis of tumor genetic heterogeneity in uveal melanoma patient-derived xenografts. Pigment Cell Melanoma Res..

[26] Popovic M., Mazzega E., Toffoletto B., de Marco A. (2018). Isolation of anti-extra-cellular vesicle single-domain antibodies by direct panning on vesicle-enriched fractions. Microb. Cell Fact..

[27] Yilmazer I., Abt M.R., Liang Y., Seung D., Zeeman S.C., Sharma M. (2023). Determining Protein-Protein Interaction with GFP-Trap Beads. Methods Mol. Biol..

[28] Caussinus E., Affolter M. (2016). deGradFP: A System to Knockdown GFP-Tagged Proteins. Methods Mol. Biol..

[29] Djender S., Schneider A., Beugnet A., Crepin R., Desrumeaux K.E., Romani C., Moutel S., Perez F., de Marco A. (2014). Bacterial cytoplasm as an effective cell compartment for producing functional VHH-based affinity reagents and *Camelidae* IgG-like recombinant antibodies. Microb. Cell Fact..

[30] Díaz D.F., Ortizz E., Martín D., Nibot C., Rizo A., Silva E. (2012). HIV-2 antibody detection after indeterminate or negative HIV-1 Western blot in Cuba, 2005–2008. MEDICC Rev..

