# Supplementary material for: Native Agarose Gels and Contact Blotting as Means to Optimize the Protocols for the Formation of Antigen–Ligand Complexes
Source: Bioengineering (Basel). 2023 Sep 22;10(10):1111. doi: 10.3390/bioengineering10101111 (PMC10604731; doi:10.3390/bioengineering10101111)
Supplement: Supplementary file 1 [file bioengineering-10-01111-s001.zip › bioengineering-2589526-supplementary.pdf]

Supplementary Material

Figure S1. Detection of ACA-SC covalent complex obtained after glutaraldehyde treatment

a) ACA and SC underwent electrophoresis either separately or together in native agarose gel, then were transferred to nitrocellulose membrane by contact blotting and stained with Ponceau S solution before being imaged using anti-HisTag-HRP antibodies; b) the covalent complex between ACA and SC was induced by glutaraldehyde addition and the reaction was stopped after 2, 5, 10 min.

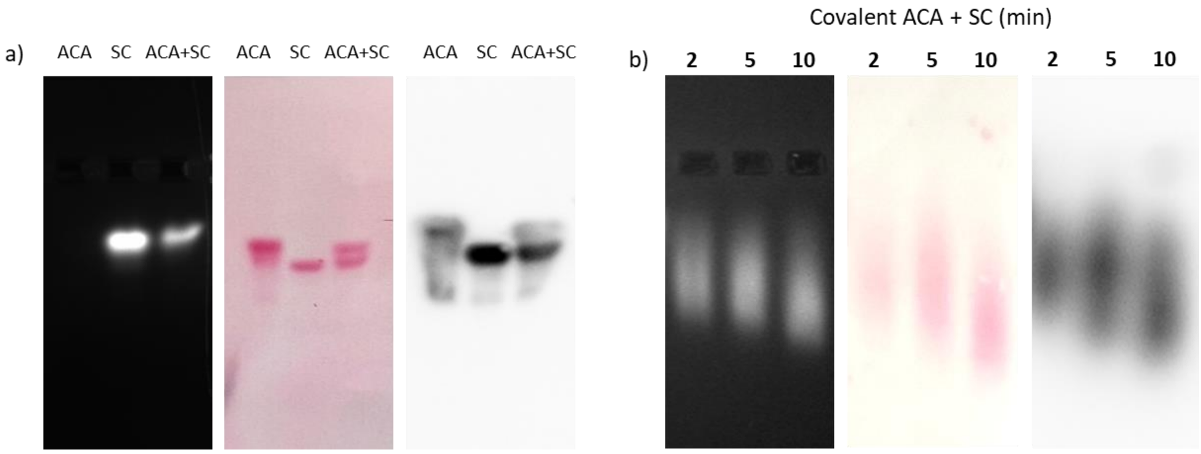

Figure S2. Buffer optimization for DSS-dependent protein complex cross-linking

The DSS-dependent formation of covalent interactions between SC and ACA was evaluated in the absence of cross-linker (nc) and in the presence of 1 mM DSS using 10 µg of the reagents (1:1 in terms of molarity) resuspended in either PBS or HEPES. Samples underwent electrophoresis (a), contact blotting and Ponceau S staining (b), and WB using anti-HisTag-HRP antibodies.

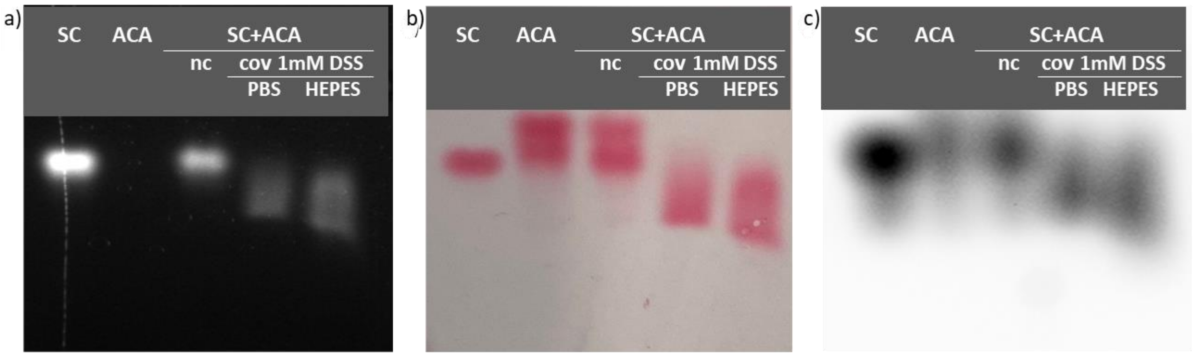

Figure S3. Detection of APEX peroxidase in native agarose gel

SC, ACA and their complex underwent electrophoresis in the absence (nc) or presence (cov) of glutaraldehyde used as a cross-linker. UV transilluminator was used to detect the fluorescent mClover3 moiety of SC (a), then the native agarose gel was incubated in TMB (APEX substrate) but no apparent enzymatic activity was detected after glutaraldehyde treatment (b).

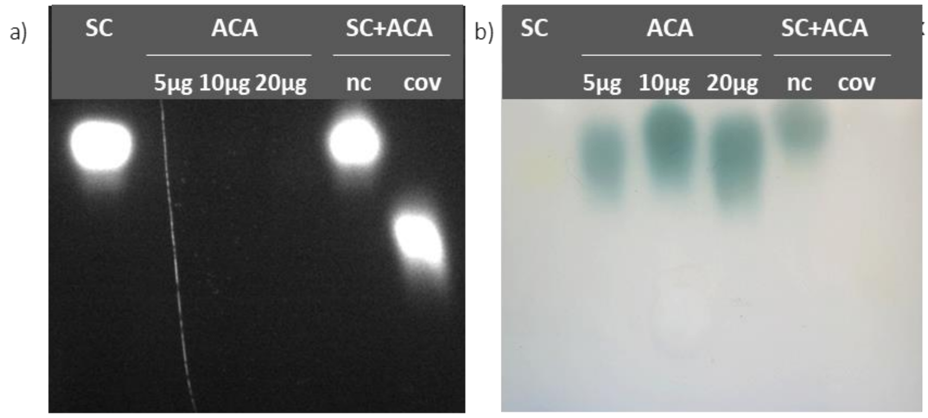

Figure S4. Quantitative evaluation of APEX activity after its blotting on nitrocellulose membrane

(a) Fluorescence signal; (b) Blotted membrane stained with Ponceau S; c) TMB staining; d) Membrane spots corresponding to SC, ACA and their 1:1 complex (non-covalent and covalent) were incubated with either TMB or Amplex®UltraRed and APEX activity was inferred by measuring spectrophotometrically the absorbance at 450 nm (TMB experiments) and the fluorescence at 595 nm (Amplex®UltraRed experiments).

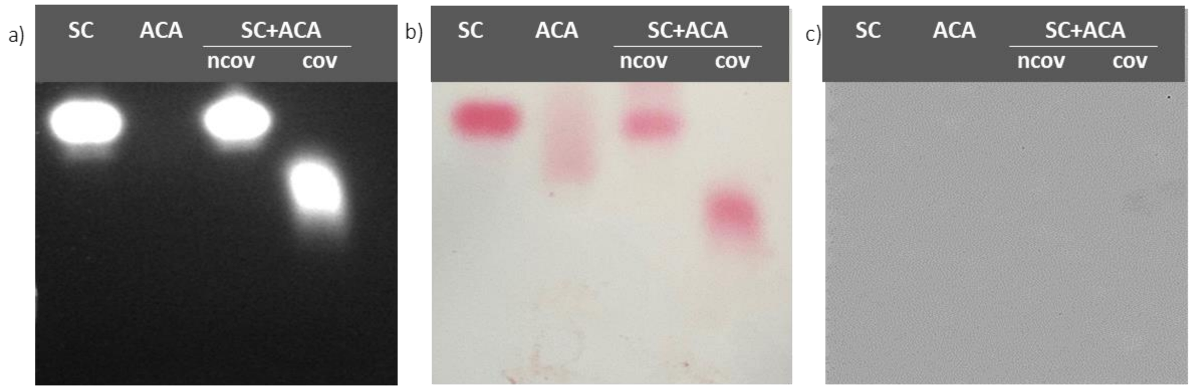

d)

Constructs	ACA	ACA+SC	SC
Fluorescence (595 nm)	38,537	32,457	196
Absorbance (450 nm)	0.101	0.105	0.005
